# The influence of elastic orthotic belt on sagittal profile in adolescent idiopathic thoracic scoliosis: a comparative radiographic study with Milwaukee brace

**DOI:** 10.1186/1471-2474-11-219

**Published:** 2010-09-23

**Authors:** Jun Jiang, Yong Qiu, Saihu Mao, Qinghua Zhao, Bangping Qian, Feng Zhu

**Affiliations:** 1 Spine Surgery, the Affiliated Drum Tower Hospital of Nanjing University Medical School, Nanjing, China

## Abstract

**Background:**

The effectiveness of bracing on preventing curve progression in coronal plane for mild and moderate adolescent idiopathic scoliosis (AIS) patients has been confirmed by previous radiographic researches. However, a hypokyphotic effect on the sagittal plane has been reported by a few studies. A relatively increasing number of AIS patients were noticed to wear a new kind of elastic orthotic belt for the treatments of scoliosis without doctors' instructions. We postulate the correcting mechanism of this new appliance may cause flattening of the spine. To our knowledge, no study has investigated the effects of this new orthosis on the sagittal profile of AIS patients. The aim of this study was to evaluate and compare the effects of elastic orthotic belt and Milwaukee brace on the sagittal alignment in AIS patients.

**Methods:**

Twenty-eight female AIS patients with mild or moderate thoracic curves were included in this study. Standing full-length lateral radiographs were obtained in three conditions: natural standing posture without any treatment, with elastic orthotic belt and with Milwaukee brace. Thoracic kyphosis (TK), lumber lordosis (LL) and pelvic incidence (PI) were measured and compared between the above three conditions.

**Results:**

Both elastic orthotic belt and Milwaukee brace can lead to significant decrease of TK, however, the decrease of TK after wearing elastic orthotic belt is significantly larger than that after wearing Milwaukee brace. Compared with no treatment, LL was found to be significantly smaller after wearing Milwaukee brace, however, such significant decrease was not noted after wearing elastic orthotic belt. No significant changes were observed for the PI between 3 conditions.

**Conclusions:**

The elastic orthotic belt could lead to more severe thoracic hypokyphosis when compared with Milwaukee brace. This belt may not be a suitable conservative method for the treatment of mild and moderate AIS patients.

## Background

**Figure 1:**
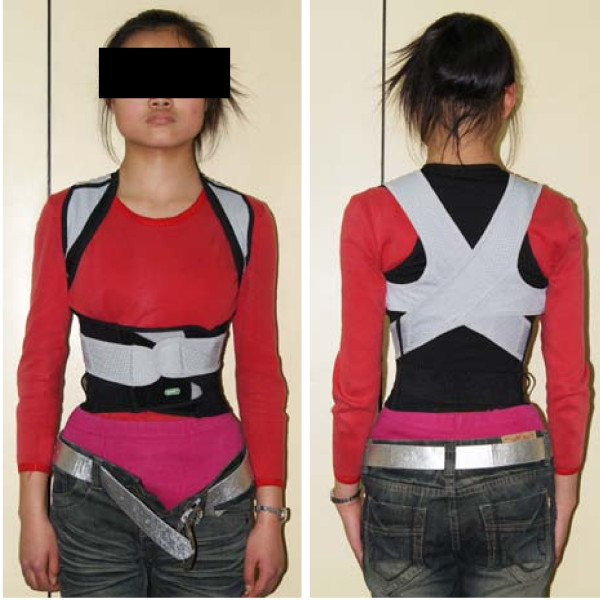
**A 14-year-old female patient wearing elastic orthotic belt**. Two straps twine around the bilateral shoulder joints to the back, then cross on the back and further extend to the front of the trunk.

Adolescent idiopathic scoliosis (AIS) is a three-dimensional spinal deformity affecting 1%-3% teenagers [1,2]. For skeletally immature patients with mild or moderate curves, bracing has been demonstrated to be the most effective conservative treatment in preventing curve progression as well as preserving the growth potentiality of spine [3-8]. Since 1995, many AIS patients with mild or moderate curve were found to wear a new kind of elastic orthotic belt in mainland China without doctors' instructions. The elastic belt is made of elastic material and mainly composed of two straps on two sides. The two straps twine around the bilateral shoulder joints to the back, then cross on the back and further extend to the front of the trunk (Figure 1). As a result, the belt produces an extension force on the thoracic cage (Figure 1). The extension force is originally supposed to correct unhealthy standing and sitting postures of adolescents, such as humpback and trunk tilt. However, it was also misused by many parents who mixed the back asymmetry of AIS up with unhealthy standing and sitting postures. Some patients preferred the elastic orthotic belt even after they were prescribed to start standard brace treatment by doctors, for this new appliance is more flexible and comfortable than rigid brace, and also for the manufacturer publicly advertised its effectiveness on correcting scoliosis.

The main purpose of bracing for AIS patients is to correct curve in frontal plane. Although it has been proved that the curve can be improved in the coronal plane by brace treatment, several studies had reported a thoracic hypokyphotic effect of bracing on the sagittal plane [9-11]. The authors postulate that elastic orthotic belt may also cause flattening of the spine since it forcedly keeps the patient in an "over-extension" position. The correcting mechanism of the belt relies on the functions of the straps on two sides which can produces an extension force on the thoracic cage and may create the effects of thoracic hypokyphosis. Decreased thoracic kyphosis is considered to be associated with curve progression and pulmonary impairment in AIS patients [12-14]. In addition, pelvic profile has been thought to influence the sagittal balance, and a larger pelvic incidence is believed to be a risk factor for the development of AIS [15]. Because a relatively large number of AIS patients wear elastic orthotic belt, and abnormal sagittal profile exerts some potential adverse effects on AIS patients, it is of great clinical importance to investigate the effects of this new elastic belt on the sagittal plane in AIS patients. The purpose of this study is to evaluate the changes of spinopelvic profile in the sagittal plane after wearing elastic orthotic belt and Milwaukee brace.

## Methods

### Subjects

**Figure 2:**
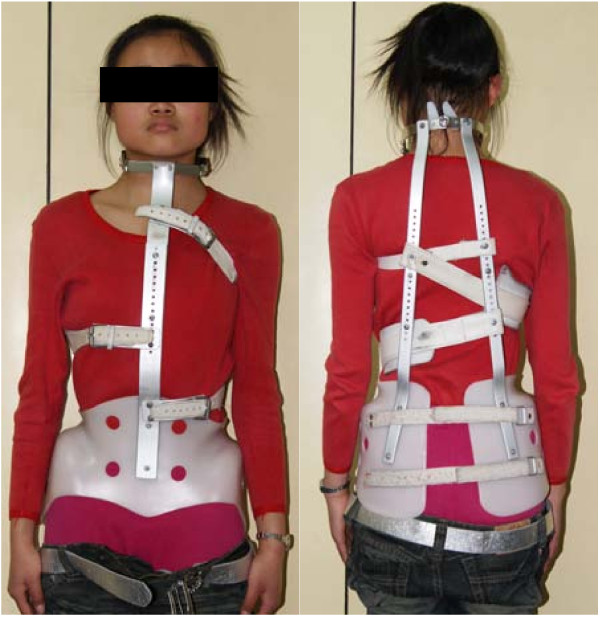
**The same patient wearing Milwaukee brace**. The neck ring cause a stimulant effect on the mandible, and two contact pads are located on the posterolateral part of the thoracic cage.

A total of 28 female thoracic AIS patients were included in this study from December, 2008 to January, 2010. All these subjects underwent careful clinical and radiologic examinations to confirm the diagnosis of AIS. They were new patients without any previous treatment and were prescribed to start standard Milwaukee brace treatments in our institution (Figure 2). All these patients had single structural thoracic curves. The average age of the patients was 14.0 years (range 12-16 years), the average Risser sign was 1.8 grades (range 0-4 grade), and the average Cobb angle was 29.6° (range 20°-44°). The apical vertebra was located in T7 in 6 patients, T8 in 11 patients and T9 in 11 patients (average 8.2). The upper end vertebra was located in T4 in 5 patients, T5 in 11 patients and T6 in 12 patients (average 5.3). The lower end vertebra was located in T10 in 4 patients, T11 in 14 patients and T12 in 10 patients (average 11.2). Two patients were before menarche. The average post-menarche age of the other 26 patients were 12.4 months (range 1~25 months). Informed consents were obtained from all the subjects and their parents. The study had been approved by the Clinical Research Ethics Committee of the hospital.

### Radiographic Evaluation

**Figure 3:**
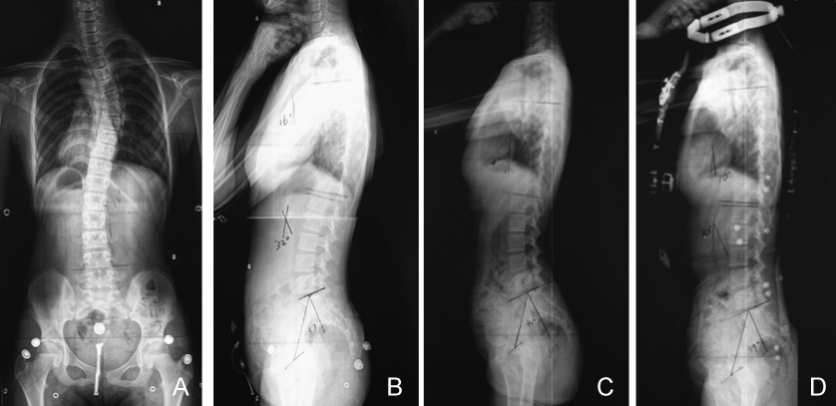
**The radiographs of the above patient A: The patient had a major thoracic curve of 35°**. The Risser sign is 3 degrees. The post-menarche age was 11 months. B: in natural standing posture, TK: 16°, LL: 32°, PI: 39°. C: with elastic orthotic belt, TK: 4°, LL: 32°, PI: 40°. D: with Milwaukee brace, TK: 10°, LL: 30°, PI: 39°.

Long-cassette standing upright lateral radiographs of the spine and pelvis [16] were obtained from each patient in 3 different conditions (Figure 3): in natural standing posture without any brace, with elastic orthotic belt and with Milwaukee brace. Three parameters in the sagittal plane were measured on each radiograph: 1) thoracic kyphosis (TK, measured between the upper endplate of the T5 and the lower endplate of the T12); 2) lumbar lordosis (LL, measured between the upper endplate of the L1 and the upper endplate of the S1); 3) pelvic incidence (PI, the angle between the perpendicular to the sacral plate midpoint and the axis of the femoral heads) [15]. The TK was considered to be negative if the curve was lordotic, and positive if the curve was kyphotic. All the patients had worn neither elastic orthotic belt nor Milwaukee brace before taking the X-ray film examinations to eliminate the potential long-term residual effects of the orthosis.

### Statistics

The data were analyzed using SPSS 13.0 statistical software (SPSS Inc., Chicago, IL). Paired-samples *t *tests were used to compare the sagittal parameters before and after wearing elastic orthotic belt, as well as before and after wearing Milwaukee brace. Independent-samples *t *tests were performed to compare changed sagittal parameters after wearing elastic orthotic belt and that after wearing Milwaukee brace. A *P *value < 0.05 was considered to be significant.

## Results

Table 1 illustrated the differences of sagittal profile between 3 conditions. The average TK was 18.2° ± 10.6° before wearing any appliance, 7.8° ± 7.2° after wearing elastic orthotic belt, and 14.7° ± 8.7° after wearing Milwaukee brace. The average decreases of TK were 10.4° after wearing elastic orthotic belt and 3.5° after wearing Milwaukee brace. Both elastic orthotic belt and Milwaukee brace caused significant decrease of TK (*P *< 0.05). TK was even significantly smaller after wearing elastic orthotic belt than that after wearing Milwaukee brace (*P *< 0.05). The mean LL was 45.1° ± 10.4° before wearing any appliance, 44.3° ± 9.5° after wearing elastic orthotic belt and 42.1° ± 9.7° after wearing Milwaukee brace. Significant decrease of LL was only found between no treatment and Milwaukee brace with a mean change of 3.0° (*P *< 0.05). The average PI was 41.0° ± 7.6° before wearing any appliance, 41.8° ± 7.9° after wearing elastic orthotic belt and 41.6° ± 7.1° after wearing Milwaukee brace. There was no significant difference of PI between 3 conditions.

**Table 1:** Comparison of sagittal parameters between 3 conditions

	In natural standing posture	with elastic orthotic belt	with brace
TK	18.2° ± 10.6°	7.8° ± 7.2°*^Δ^	14.7° ± 8.7°*
LL	45.1° ± 10.4°	44.3° ± 9.5°	42.1° ± 9.7°*
PI	41.0° ± 7.6°	41.8° ± 7.9°	41.6° ± 7.1°

* means significant difference between brace (or elastic orthotic belt) and natural standing posture (p < 0.05).

Δ means significant difference between brace and elastic orthotic belt (p < 0.05).

## Discussion

The abnormal sagittal alignment has been implicated to be related to the development and progression of AIS [15,17,18]. The thoracic kyphosis is normally protected from buckling by being behind the axis of spinal column rotation [19]. However, previous radiological studies have demonstrated the phenomenon of thoracic hypokyphosis in AIS patients which is considered to be caused by disproportionate growth of the anterior and posterior spinal columns [15,20,21]. Thoracic hypokyphosis may bring the apical region of thoracic spine anterior to the axis of spinal column rotation and finally cause axial rotational instability under compression [22]. A positive correlation between the severity of the thoracic curve and the ratio of differential growth between the anterior and posterior columns has been reported by a MRI study [17]. Rigo [14] also found that the patients with smaller thoracic kyphotic angles had more severe thoracic curves. Taken together, these data suggested that thoracic hypokyphosis may be a risk factor for thoracic curve progression. Several studies also suggested a strong correlation between the degree of thoracic hypokyphosis and the decrease of pulmonary function in AIS patients [23-25]. Upadhyay [23] found that thoracic hypokyphosis had a significant effect on the decrease of lung volumes. Kearon [25] also found that the thoracic hypokyphosis was an important factor contributing to pulmonary impairment. In a large sample study, AIS patients with thoracic hypokyphosis were more likely to have moderate and severe pulmonary damages compared with those having normal thoracic kyphotic angles [24]. The thoracic kyphosis was found to be an independent predictor of reduced pulmonary impairment by stepwise multiple regression analysis [24].

The major correcting mechanism of bracing is to apply external forces on subcutaneous skeletal structures, such as rib cage, which are attached to vertebrae rigidly [19,26]. Lateral external forces are applied on the apical vertebra and contralateral forces are applied above and below the apical vertebra. These forces can produce a "three-points" effect which leads to the coronal curve correction [19]. Meanwhile, the brace could also exert external forces on the back which cause the flattening of the spine [19]. The Milwaukee brace could lead to decreased TK due to the force of contact pad on back of thoracic cage theoretically. However, there were few studies about the influence of Milwaukee brace on the sagittal profile of spine in AIS patients.

In recent years, a new kind of elastic orthotic belt has been commercialized in mainland China for the treatment of so-called "unhealthy" sitting and standing postures commonly seen in Chinese adolescents. These teenagers show mild hump back due to long-time paper work on the desk with unhealthy postures. The elastic orthotic belt is designed with the intention to straight up the spine and to correct the hump back by two tension straps. Presently, a relative large number of AIS patients are receiving orthotic belt treatment due to the parents' confusion of cosmetic appearance of early AIS and unhealthy sitting posture as well as the manufacturer's promotion in various media. The authors hypothesized that the tension straps could result in thoracic hypokyphosis in AIS patients for they could provide "over-extension" tensile forces on the thoracic cage. Given the potential adverse influences of thoracic hypokyphosis on the curve progression and pulmonary function impairment, the change of the sagittal profile after wearing elastic orthotic belt draws our great interests.

In the present study, the elastic orthotic belt resulted in significant decrease of TK with an average decrease of 10.4°. Milwaukee brace could also cause significant reduction of TK with an average decrease of 3.5°. The minor average decrease of TK after wearing Milwaukee brace is also statistically significant because of the powerful efficacy of paired-*t *test. It is noticed that the elastic orthotic belt can lead to significantly more severe thoracic hypokyphosis when compared with Milwaukee brace. The current study also demonstrated that the Milwaukee brace could lead to a significantly smaller LL while the LL is not significantly changed after wearing elastic orthotic belt. We postulate that the significantly decreased LL with Milwaukee brace may be mainly caused by the stimulant effect of the neck ring on the mandible, which results in the reflection of "upward extension" of the trunk. The effect of this "upward extension" could act as "pull strength" which also leads to the straightening of the spine.

Different from standard scoliotic brace, the designing rationale of this elastic orthotic belt is not consistent with the mechanism of coronal curve correction. This belt can not apply lateral forces on proper contact point for deformity correction. Another kind of elastic orthosis named "SpineCor" developed by Coillard and Rivard has been used for the treatments of AIS patients in past years [27]. However, the failure rate of the SpineCor was significantly higher than that of the conventional brace. The lower success rate of SpineCor suggested that elastic orthosis may not be a suitable conservative treatment for AIS patients. We highly doubt the effectiveness of the elastic belt in controlling curve progression for three main reasons: 1) the thoracic hypokyphotic effect may contribute to the curve progression, 2) the design principle is not consistent with the mechanism of curve correction, 3) soft orthotic appliance shows higher failure rate of correcting scoliosis. Besides the uselessness in correcting curves, we also presumed that elastic orthotic belt could lead to more impairment of pulmonary function because of the correlation between thoracic hypokyphosis and the pulmonary function impairment in AIS patients.

Pelvic alignment is also considered to influence the sagittal spinal balance. A representative morphological parameter, pelvic incidence (PI), is significantly larger in AIS patients when compared with normal adolescents [15]. The increased PI could be a result of the overall altered sagittal alignment in AIS patients and is also believed to be a risk factor for curve progression [15]. No significant differences of PI between three conditions were found in our study. It is concluded that neither brace nor elastic belt has any effects on the pelvic profile in AIS patients. In fact, the PI is a relatively stable pelvic parameter of each individual and unaffected by the change of sagittal profile [28]. Our results of unchanged PI in 3 different conditions fully support this view.

Several limitations should be mentioned in the current study. First, although all the patients had not worn any kind of brace before receiving the X-ray film examinations, they do wear elastic belt before wearing Milwaukee brace. Hence, the potential immediate residual effect of elastic belt was not taken in account. Second, because increased radiation exposure may pose more adverse influences on the immature AIS patients, we did not investigate the effects of elastic orthotic belt and Milwaukee brace on the coronal plane. Third, as an ethical consideration, no patients included in this study received elastic orthotic belt treatment. Therefore, the success rate of brace and that of elastic orthotic belt can not be compared to evaluate the effectiveness of these two methods. However, the authors still insist that the elastic orthotic belt is not suitable to be used as a conservative method for AIS patients because of its hypokyphotic effects on thoracic spine and its "soft" correcting forces.

## Conclusion

Although Milwaukee brace leads to significant decreased TK in AIS patients, the change of TK is small. Elastic orthotic belt could result in a more severe thoracic hypokyphosis compared with Milwaukee brace. Given the possible adverse influence of the elastic orthotic belt on AIS patients, the authors suggest that borderline cases of AIS should undergo careful clinical and radiologic examinations to confirm the diagnosis, and receive standard bracing if conservative treatment is advised. Wearing elastic orthotic belt may not be a suitable nonsurgical treatment for AIS patients.

## Competing interests

The authors declare that they have no competing interests.

## Authors' contributions

All the authors contributed to the design, data collection and analysis of this investigation. All the authors have read and approved the final manuscript.

## Pre-publication history

The pre-publication history for this paper can be accessed here:

http://www.biomedcentral.com/1471-2474/11/219/prepub
